# Supplementary material for: What does it take to learn the rules of RNA base pairing? A lot less than you may think
Source: bioRxiv. 2025 Aug 2:2025.07.31.668042. Preprint. [Version 1] doi: 10.1101/2025.07.31.668042 (PMC12324431; doi:10.1101/2025.07.31.668042)
Supplement: Supplement 1 [file media-1.gz › supplemental_material/experiments/g6_optimize_param_conus_rnabench_RNaseP_random/losses_i5.pdf]

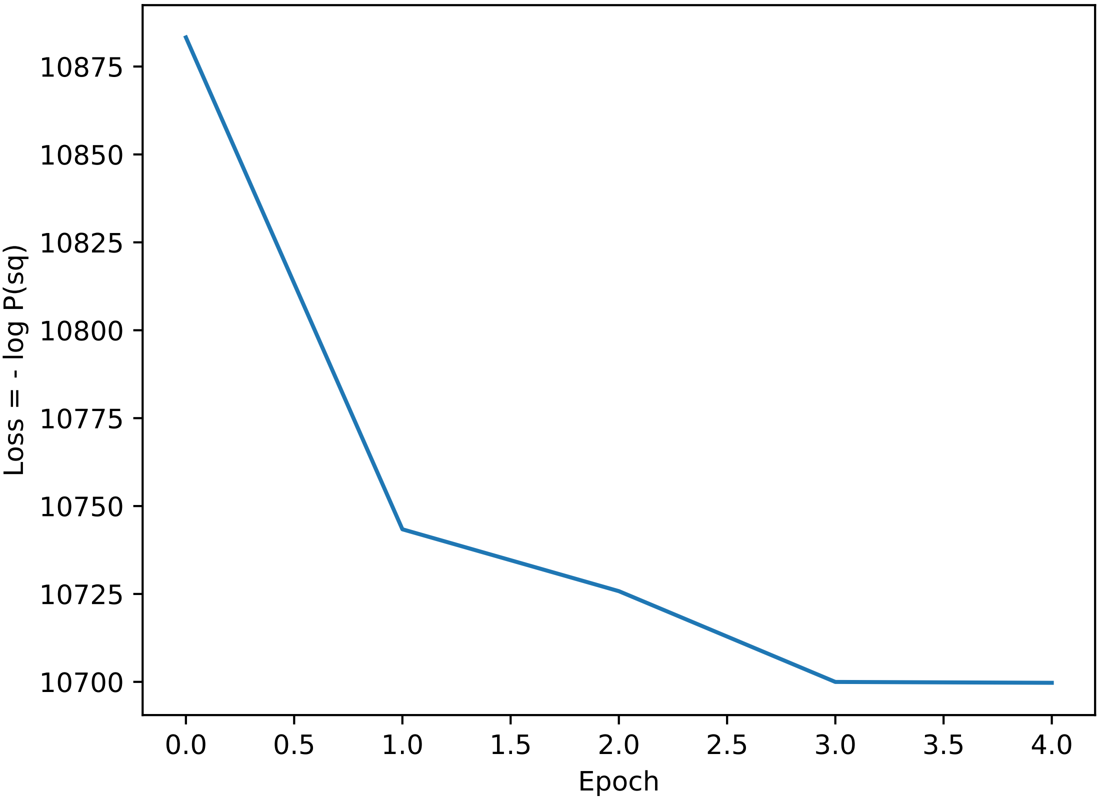

Loss = - log P(sq)
10875
10850
10825
10800
10775
10750
10725
10700
0.0
0.5
1.0
1.5
2.0
2.5
3.0
3.5
4.0
Epoch
